# Biomarkers for Colorectal Cancer

**DOI:** 10.3390/ijms11093209

**Published:** 2010-09-13

**Authors:** Takuji Tanaka, Mayu Tanaka, Takahiro Tanaka, Rikako Ishigamori

**Affiliations:** 1 The Tohkai Cytopathology Institute: Cancer Research and Prevention (TCI-CaRP), 5-1-2 Minami- Uzura, Gifu 500-8285, Japan; 2 Department Oncologic Pathology, Kanazawa Medical University, 1-1 daigaku, Uchinada Ishikawa 920-0293, Japan; 3 Department of Pharmacy, Kinjo Gakuin University of Pharmacy, Moriyama-Ku, Nagoya, Aichi 463-8521, Japan; 4 Department of Physical Therapy, Kansai University of Health Sciences, Kumatori-Machi, Sennan-Gun, Osaka 590-0482, Japan; E-Mail: tmntt08@gmail.com; 5 Cancer Prevention Basic Research Project, National Cancer Center Research Institute, Tokyo 104-0045, Japan; E-Mail: rishigam@ncc.go.jp

**Keywords:** biomarkers, colorectal cancer, fecal biomarkers, genomic and epigenetic biomarkers, serum biomarkers, microRNA

## Abstract

Colorectal cancer (CRC) is the third most common epithelial malignancy in the world. Since CRC develops slowly from removable precancerous lesions, detection of the lesion at an early stage by regular health examinations can reduce the incidence and mortality of this malignancy. Colonoscopy significantly improves the detection rate of CRC, but the examination is expensive and inconvenient. Therefore, we need novel biomarkers that are non-invasive to enable us to detect CRC quite early. A number of validation studies have been conducted to evaluate genetic, epigenetic or protein markers for identification in the stool and/or serum. Currently, the fecal occult blood test is the most widely used method of screening for CRC. However, advances in genomics and proteomics will lead to the discovery of novel non-invasive biomarkers.

## 1. Introduction

Various types of cancer biomarkers are listed in Table 1.

Any measurable specific molecular alteration of a cancer cell either at the DNA, RNA, protein, or metabolite level can be referred to as a cancer biomarker. The expression of a distinct gene can enable its identification in a tissue in which none of the surrounding non-cancerous cells express the specific marker. It is difficult to distinguish related disease subtypes that have different clinical outcomes. There is therefore a need for more exact molecular biomarkers for use in clinical practice. Recently, the discovery of cancer biomarkers has become a major focus of cancer research and there are thousands of publications on cancer biomarkers. The ideal biomarkers for cancer have applications in determining predisposition, early detection, assessment of prognosis, and drug response. The biomarker that serves as a target for drug development would have an additional advantage. Desirable characteristics of molecular markers for cancer are postulated, but no biomarker meets these ideal characteristics. Hence, there is an urgent need for cancer biomarkers with more accurate diagnostic capability, particularly for early-stage cancer.

Colorectal cancer (CRC) is the third most common malignancy in the world. In addition, there are approximately 1,000,000 new cases of CRC and 500,000 deaths associated with CRC each year. Indeed, CRC represents one of the primary causes of cancer deaths in Europe and the United States [1]. In Asia, including Japan, CRC is the fourth leading cause of mortality by cancer, and its incidence is increasing [2]. CRC develops slowly via a progressive accumulation of genetic mutations. Therefore, the risk of recurrence and subsequent death due to CRC is closely related to the stage of the disease at the time of the first diagnosis. Recent studies have shown that shifting the detection of the disease to an earlier stage via mass screening and intervening at early stage can reduce the risk of death from CRC [3,4]. These findings thus suggest the clinical need for biomarkers for early detection of CRC.

Biomarkers are used as indicators of a biological state of tissues. Therefore, biomarkers have characteristics that enable them to be objectively measured and evaluated as indicators of normal biological processes, pathogenic processes, or pharmacologic responses to a therapeutic intervention. One of the key requirements of biomarkers for detecting CRC is that it must allow detection of the disease at earlier stages. Such tests using biomarkers should have high sensitivity and specificity, while producing a low number of false-negative and false-positive results, to prevent subjecting healthy individuals to unnecessary colonoscopies. Colonoscopy offers significant improvements in the detection rates for CRC, but the diagnostic value of this is limited in relation to costs, risks, and inconvenience [5]. Non-invasive biomarkers have the potential to greatly enhance screening acceptance. Several non-invasive tests for detecting CRC are available, of which the fecal occult blood test (FOBT) is the most commonly used [6,7]. However, this test lacks sensitivity as well as specificity for screening an average risk population. Thus, novel CRC biomarkers that will further enhance the detection of the disease and trigger follow-up colonoscopies when necessary should be developed. In addition to such detection biomarkers, prognostic markers which can predict the likely course of the cancer, stratification markers which can predict the likely response to drugs prior to beginning treatment, and efficacy markers which can monitor the efficacy of drugs treatment may also reduce the mortality rate of CRC.

Recent advances in genomics and proteomics have contributed to our understanding of the natural history of cancers. Genomic techniques, such as DNA microarray analysis and proteomic methods, for example, 2-dimensional electrophoresis and mass spectrometry, are now commonly used to evaluate the expression profiles of genes and proteins in cancer cells, their surrounding tissues, and body fluids [8]. Identification of genes and/or proteins that are characteristic of the development of cancer can potentially uncover biomarkers that will aid in the diagnosis of CRC. In this review, we will focus on potential non-biomarkers which have recently been discovered and non-invasive biomarkers which are currently being used in clinical settings (Table 2).

## 2. Fecal Markers

### 2.1. Fecal Hemoglobin

Stool-based detection of CRC is quite simple, inexpensive, and the least invasive method of screening available [9]. FOBT detecting hemoglobin enzymatically or immunologically is the most widely used screening modality for CRC and [10]. Enzymatic FOBT measures the peroxidase-like activity of hemoglobin originating from any source. Therefore, enzymatic FOBT is susceptible to bleeding from both colorectal and upper gastrointestinal tracts. In addition, the ingestion of certain foods (red meats, fruits and vegetables) and medicines (non-steroidal anti-inflammatory drugs) led also to false-positive results. Immunological FOBT using antibodies which specifically detect human hemoglobin is not impacted by plant peroxidase in the diet. An important limitation of the FOBT is the relatively poor sensitivity at detecting early-stage lesions. Low sensitivity of the FOBT for the detection of colorectal neoplasms is reported to be ~10% of adenomas and 40~85% of CRCs. In fact, randomized clinical trials indicated that FOBT is not very reliable and that it only reduces CRC mortality by 30% [3,11].

### 2.2. Genes and Epigenetic Markers

Cryptal cells (colonocytes) are shed into the fecal stream and provide informative materials that can be used to detect genes and epigenetic markers in feces [12]. Unlike fecal blood, cryptal cells are shed continuously. Furthermore, the shedding of cancer cells from CRC occurs more frequently than from normal colonic epithelium. Fecal cells including cancer cells can be assessed by analyzing DNA mutations for targets such as K-*ras*, *p53*, and adenomatous polyposis coli (*APC*), by analyzing epigenetic markers such as microsatellite instability (MSI), or by measuring unfragmented long-form DNA (L-DNA).

K-*ras*, encoding a Ras family protein, functions as a guanine nucleotide binding protein that is involved in a signal transduction pathway including the phosphatidylinositol-3-kinase and serine/threonine protein kinase B pathways [13]. K-*ras* mutations are found in 40–50% of sporadic colon cancers and adenomas [14]. In addition, K-*ras* mutations being present in aberrant crypt foci, which are putative pre-cancerous lesions, are reported to be found in 13–95% of CRC [15–18]. Thus, K-*ras* mutations might be an important early event in colorectal carcinogenesis.

*p53* encoding a tumor suppressor protein which regulates the expression of genes involved in apoptosis, angiogenesis, the cell cycle and maintenance of the genome [19]. Approximately half of human cancers contain mutated *p53* genes, and 30–60% of CRCs have mutations in the gene [20]. The mutations depend on the stage, grade and location of the cancer. The mutations appear to be at relatively late stage of colorectal carcinogenesis, and altered p53 has only modest impact on the outcome of CRC. Therefore, the relatively low mutation rate of p53 at early-stage limits the use in DNA-based detection of CRC.

APC protein is another tumor suppressor which assembles on a scaffold protein, axin, with β-catenin and glycogen synthase kinase 3β to coordinate the regulation of β-catenin signaling [21]. Inactivation of the APC protein is responsible for both inherited and sporadic types of CRC. Like K-*ras*, *APC* mutation appears to be an early-genetic event during the progression from adenoma to adenocarcinoma, suggesting its potential for use as a screening biomarker. However, unlike K-ras, the mutations are distributed throughout the coding region, thereby making it technically difficult and time consuming to detect all of the potential mutations during screening for CRC [22,23].

Microsatellites are stretches of short DNA sequences which contain a motif of 1–5 nucleotides with tandem repeats [24]. The most common microsatellite in human DNA is a dinucleotide repeat of cytosine and adenine. These tandem repeats occur throughout the human genome. MSI occurs when microsatellites undergo changes in length. MSI is observed in approximately 15% of the CRC tumors. Tumors with MSI have better prognosis than stage-matched tumors with stable microsatellite [25]. In sporadic CRC, MSI most commonly occurs due to epigenetic silencing of the DNA mismatch repair gene, *MLH1* [26]. There are several MSI markers, among which *BAT26* is probably the most widely used.

Shedding of cryptal cells is a normal consequence of exfoliation [27]. Untransformed cryptal cells are shed continuously from the colonic mucosa. These cells usually undergo apoptosis. Conversely, malignant CRC cells shed from tumor mass have a decreased rate of apoptosis relative to normal cryptal cells, which facilitate detection of intact genomic DNA (L-DNA) as a potential stool-based marker. Boynton [28] amplified six genomic fragments of different length from each of four different genetic loci (*APC*, *p53*, *BRCA1*, and *BRCA2*) using fecal specimens collected from 25 CRC patients and 77 controls. In this study, when a positive L-DNA was defined as >18 bands detected from a possible 24 bands (4 loci × 6 fragments), the specificity for CRC detection was 97% and the sensitivity was 57%.

A large population-based study revealed that a fecal DNA panel consisting of 21 mutations (three in the K-*ras* gene, 10 in the *APC* gene, and eight in the *p53* gene; the MSI markers of *BAT-26*; and L-DNA) detects a greater proportion of CRC than FOBT without compromising specificity [29]. In addition, the sensitivity of the fecal DNA panel was 52% for invasive CRC and 41% for invasive CRC plus adenomas with high-grade dysplasia, whereas that of the FOBT was 13% for the former and 14% for the latter. In subjects with negative findings on colonoscopy, the DNA panel had a specificity of 94%, whereas the FOBT had a specificity of 95%. The results of the study clearly indicate that the DNA panel has a greater sensitivity than the FOBT without reduced specificity.

## 3. Serum or Blood Markers

### 3.1. CEA

Carcinoembryonic antigen (CEA) is a high molecular weight glycoprotein belonging to the immunoglobulin superfamily. The carboxy-terminal of CEA contains a hydrophobic region which is modified to provide a glycosyl phosphatidylinositol link to the cell membrane. While the presence can be determined in biopsy samples, it is usually identified in serum. This protein has been used for many years as a biomarker of CRC as well as cancers developing in other tissues [30]. High CEA levels are specifically associated with CRC progression, and increased levels of the marker are expected to fall following CRC surgery [31]. However, even in the absence of cancer, high CEA levels may also occur in response to inflammatory conditions, such as hepatitis, inflammatory bowel disease (IBD), pancreatitis, and obstructive pulmonary disease. In addition, CEA may not be elevated when CRC is at advanced stage. Thus, CEA does not provide sufficient sensitivity and reliability for the early detection of CRC. The potential value of the CEA test lies in its use to measure the course of the progression of cancer as a prognostic marker. CRC patients with higher CEA levels have poorer prognosis.

### 3.2. CA 19-9

Carbohydrate antigen (CA) 19-9, which is the second most investigated gastrointestinal tumor marker, is known to be a sialylated Lewis-a antigen [32]. CA 19-9 was originally defined by a monoclonal antibody produced by hybridoma prepared from the spleen cells of mice immunized with the human CRC cell line, SW 1116. Although CA 19-9 is the best marker available for pancreatic adenocarcinoma, CA 19-9 is less sensitive than CEA for CRC and also gives less information than CEA [33]. Other carbohydrate antigens, such as CA 50, CA 195, CA 242, CA M26, CA M25, CA M43 and CA 72-4, have also been evaluated extensively [34], but, due to their sensitivity, stage dependency and specificity, these antigens are not useful markers for the detection of CRC.

### 3.3. Tissue Inhibitor of Metalloproteinase Type 1

Tissue inhibitor of metalloproteinase type (TIMP)-1 is a multifunctional glycoprotein which inhibits most matrix metalloproteinases (MMPs). The total levels of TIMP-1 in patients with CRC are significantly greater when compared to that of healthy blood donors who have a very narrow range of plasma TIMP-1 levels [35,36]. More importantly, TIMP-1 is capable of being detected at early stages of CRC. Conversely, plasma levels of total TIMP-1 in patients with colonic adenomas, IBD or primary breast cancer, do not increase [37]. Preoperative TIMP-1 levels were proposed as a stage-independent prognostic biomarker for CRC in two independent studies [38,39]. The results of these studies, however, indicated that elevation of TIMP-1 was restricted to advanced stages of CRC. Additional studies are required to validate the use of TIMP-1 for both early diagnosis and evaluation of the prognosis of CRC.

### 3.4. Five-Serum-Marker Panel (Spondin-2, DcR3, Trail-R2, Reg IV, MIC 1)

Four serum biomarkers, spondin-2, tumor necrosis factor receptor superfamily member 6B (DcR3), TRAIL receptor 2 (TRAIL-R2) and Reg IV were recently evaluated in 600 serum samples. All four markers, as well as a fifth marker, macrophage inhibitory cytokine 1 (MIC1), were elevated in patients with CRC when compared to normal controls and patients with benign diseases. Additionally, this five-serum biomarker panel may have better sensitivity and specificity than CEA to improve the detection rate of early stage CRC.

### 3.5. Nicotinamide N-methyltransferase and Proteasome Activator Complex Subunit 3

To analyze 16 matched CRC and adjacent normal tissue samples, two-dimensional gel electrophoresis and mass spectrometry were used. Then proteins found to be elevated in cancer tissue were further validated with serum samples. Elevated levels of nicotinamide N-methyl-transferase (NNMT) and proteasome activator complex subunit 3 (PSME3), which are not predicted to be secreted, were found in serum from patients with CRC [40,41]. Validation studies using 109 CRC samples, 317 healthy control samples, and 87 samples from patients with benign large bowel diseases revealed that the diagnostic accuracy of PSME3 was similar to that of CEA, and that NNMT was better than CEA at detecting CRC.

### 3.6. Collapsin Response Mediator Protein-2

By analyzing the secretomes of 21 cancer cell lines derived from 12 cancer types, collapsin response mediator protein-2 (CRMP-2) was identified to be a potential CRC biomarker in the serums of 201 CRC patients and 210 healthy controls [42]. The use of CRMP-2 alone showed better sensitivity, but poorer specificity than CEA. Combined detection using CEA and CRMP-2 however produced better sensitivity (77%) and specificity (95%) than detection using either of these markers alone (43 and 61% sensitivity, respectively; 87 and 65% specificity, respectively). Thus, CRMP-2 might be a valuable serum marker when used in combination with CEA.

## 4. MicroRNA

For the past decade, the development of genomic technology has revolutionized modern biological research and drug discovery. Functional genomic analyses enable biologists to perform analysis of genetic events on a global scale and they have been widely used in gene discovery, biomarker determination, disease classification, and drug target identification. In this article, we provide an overview of the current and emerging tools involved in genomic studies, including expression arrays, microRNA (miRNA) arrays, array CGH, ChIP-on-chip, methylation arrays, mutation analysis, genome-wide association studies, proteomic analysis, integrated functional genomic analysis and related bioinformatic and biostatistical analyses [43].

Post-transcriptional regulation of gene expression by miRNA has recently attracted major interest among cancer researchers in relation to its involvement in cancer development. More than 1000 miRNAs are expressed in human cells, some tissue or cell type specific, others considered as house-keeping molecules. Functions and direct mRNA targets for some miRNAs have been relatively well studied over the last years. Every miRNA potentially regulates the expression of numerous protein-coding genes (tens to hundreds), but it has become increasingly clear that not all miRNAs are equally important; diverse high-throughput screenings of various systems have identified a limited number of key functional miRNAs over and over again. Particular miRNAs emerge as principal regulators that control major cell functions in various physiological and pathophysiological settings [44]. Altered miRNAs are reported in cancers of several tissues, including colon [45–47], liver [48], prostate [49], esophagus [50], brain [51], pancreas [52], breast [53], and chronic degenerative disease [54].

As to CRC, Dr. Nakagama’s group [55,56] has demonstrated that Staphylococcal nuclease homology domain 1 (SND1), a component of RISC, is frequently up-regulated in human colon cancers and also chemically-induced colon cancers in animals, as well as in preneoplastic lesions of the colon. Overexpression of SND1 in colon cancer cells caused down-regulation of APC and activation of the Wnt signaling pathway as a consequence, without altering APC mRNA levels. Post-transcriptional regulation of gene expression by SND1 was suggested to be mediated by miRNA through the 3′-UTR containing the miRNA target sequence. As for the miRNA expression profile, miR-34a was among the list of down-regulated miRNA in human colon cancer, suggesting its tumor suppressive role in colon carcinogenesis. Expression of miR-34a is tightly regulated by p53, and ectopic expression of miR-34a in colon cancer cells causes remarkable reduction of cell proliferation and induces senescence-like phenotypes. miR-34a also down-regulates silent information regulator 1 (SIRT1), which is a class III histone deacetlylase and known to be a negative regulator of p53 through the modulation of acetylation at K382 of p53, and participates in the positive feedback loop of the p53 tumor suppressor network. Other investigators [57] also suggested tumor suppressor functions of miR-34a, in part, through a SIRT1-p53 pathway. Recently, miRNA expression in CRC has been found to be associated with microsatellite instability (MSI) subgroups, including low MSI and HNPCC-associated cancers [58]. Thus, miRNA are potential diagnostic and prognostic markers (Table 3), as well as therapeutic targets for CRC.

## 5. Other Potential Biomarkers

Habermann demonstrated that C3a-desArg is present at significantly higher levels in serum from patients with colorectal adenomas and carcinomas than in serum from healthy individuals [75]. Specifically, in a blinded validation study (n = 59), the use of C3a-desArg alone predicted the presence of CRC with a sensitivity of 97% and a specificity of 96%.

Analyzing the protein profiles of colon cancer serum and protein profiles of CRC tumors demonstrated that human neutrophil peptides (HNP)-1, HNP-2 and HNP-3, also known as a-defensin-1, adefensin-2, and a-defensin-3, are up-regulated in CRC patients [76.^77^]. Indeed, the HNP1–3 level in the serum of 48 CRC patients and 42 normal controls was capable of identifying CRC with a sensitivity of 69% and a specificity of 100%.

The gene expression level of macrophage migration inhibitory factor (MIF) is elevated in CRC tissues, suggesting the use of the protein as a potential biomarker for CRC. In an analysis of serum samples of 129 patients with colon cancer and 53 healthy control subjects, the serum MIF level was found to be significantly increased in patients with CRC [78]. Although the specificity of MIF is not as high as that of CEA (90.6% *vs*. 100.0%), MIF is more sensitive during early cancer detection (47.3% *vs*. 29.5%), which suggests that MIF may be used as a diagnostic marker in CRC.

The serum levels of both macrophage-colony stimulating factor (M-CSF) and granulocyte-colony stimulating factor are significantly higher in CRC patients than in healthy subjects [79,80]. In addition, serum levels of M-CSF are more associated with lymph node metastasis than CEA and CA 19-9, which suggests that serum M-CSF elevation in CRC patients might help predict the risk of lymph node metastasis of this tumor. M-CSF may offer additional information to that presented by classic prognostic factors.

Prolactin that is synthesized by the anterior pituitary gland is a hormone with multiple biological actions and is elevated in patients with CRC. A study that evaluated 47 CRC patients and 51 healthy controls revealed that prolactin can predict CRC with a sensitivity and specificity of 77% and 98%, respectively [81].

M2-pyruvate kinase is an isoform of glycolytic enzyme pyruvate kinase. Although this protein is a cytosolic enzyme, it is liberated into circulation via an unknown mechanism. It is suggested that M2-pyruvate kinase is released into circulation from dying cancer cells. M2-pyruvate may thus be a useful marker for the detection of CRC. Two independent studies revealed that the use of M2-pyruvate kinase for the detection of CRC has a sensitivity of 48–58% and a specificity of 90–95%. Further, when combined with CEA, the sensitivity of M2-pyruvate increases without decreasing the specificity [82,83].

Recent evidence suggests that the assessment of epigenetic events is one of the most promising means of identifying biomarker candidates for the early detection of cancer. DNA methylation, in which cytosines within the palindromic dinucleotide 5′-CpG-3′ sequence are methylated, shapes the chromatin structure of DNA according to its functional state [84,85]. The cancer genome is frequently characterized by hypermethylation of specific genes. Therefore, epigenomics AG has developed a blood test for CRC that is based on methylation of SEPT9, NGFR and TMEEF2 [86]. The evaluation study of this test, using free-floating DNA extracted from plasma samples of 133 CRC patients and 179 healthy controls, in the same age range, to determine the methylation levels, using restriction enzyme-based qPCR, revealed that the biomarker with the highest performance was SEPT9, which was capable of detecting CRC with a specificity and sensitivity of 95% and 52%, respectively, when a cutoff of 0.011 μg/L of methylated SEPT9 DNA was used.

Gene expression patterns in the peripheral blood reflect changes that occur within the cells and tissues of the body [86]. Han [87] extracted total RNA from the white blood cells of peripheral blood and identified differentially regulated genes using a microarray. Specifically, they used a panel comprised of five genes including B-cell scaffold protein with ankyrin repeats 1 (BANKI), B-cell novel protein 1 (BCNPI), cytidine deaminase (CDS), FERM domain containing 3 (MGC20553), and membrane-spanning 4-domains, subfamily A, member 1 (MS4AI), to detect CRC. This test had a sensitivity of 88–94% and a specificity of 64–77%.

Three proteins, colon cancer-specific antigen (CCSA)-2, CCSA-3 and CCSA-4, have shown promise as markers for the detection of CRC. Using a cutoff value of 2 μg/mL for CCSA-3, both CRC and advanced adenoma were detected with 89% sensitivity and 82% specificity [88]. When CCSA-4 was used with a cutoff value of 0.3 μg/mL, the sensitivity and specificity was 85% and 91%, respectively. The use of CCSA-2 at a cutoff of 10.8 μg/mL had an overall specificity of 78% and sensitivity of 97% when used on separate individuals with advanced adenomas and CRC from normal, hyperplastic polyp, and adenoma populations [89].

Remodeling of the extracellular matrix is important in the development of epithelial malignancies, and several extracellular matrix proteins that can be liberated into circulation have been evaluated as potential biomarkers. The results of these evaluations have revealed that the serum levels of MMP9 and MMP7 depend on the presence of CRC [90,91]. In addition, serum laminin and MMP7 can be used as independent prognostic markers of CRC [91,92].

However, large scale clinical studies are required to refine and validate the diagnostic accuracy of the findings mentioned above.

## 6. Conclusions

Cancer biomarkers and characteristics of an ideal biomarker for CRC are discussed in this review, as well as technologies for their detection. The focus of this article is on the use of biomarkers for anticancer drug development and clinical applications, including determination of prognosis as well as monitoring of response to therapy. Types of biomarkers include serum/blood markers, fecal markers and miRNA. Currently, the FOBT is the only screening modality for CRC. DNA-based fecal markers are promising but are not widely used in clinical settings. In addition, a lack of sensitivity and specificity preclude the use of all existing serum markers for the early detection of CRC. CEA is used to monitor therapy in advanced CRC, and the pre-operative level of CEA is used to provide prognostic information. However, there is insufficient evidence for routine use of other classic serum markers such as carbohydrate antigens and TIMP-1. Therefore, large scale validation studies are required to evaluate the potential for the use of biomarkers that have recently been discovered through ‘–omics’ technology. Within clinical research, oncology is expected to have the largest gains from biomarkers over the next five to ten years. Development of personalized medicine for cancer is closely linked to biomarkers, which may serve as the basis for diagnosis, drug discovery and monitoring of diseases. A major challenge in development of cancer biomarkers will be the integration of proteomics with genomics and metabolomics data and their functional interpretation in conjunction with clinical data and epidemiology [93].

## Figures and Tables

**Table 1:** Cancer biomarkers.

Type of biomarkers	Analysis
Genetic	Gene mutations
	Tumor suppressor gene status
DNA	Gene amplification
	Microsatellite instability
	Mitochondrial DNA
Epigenetic	DNA methylation
RNA	microRNAs
Protein	-
Metabolic	-
Immunological	T-cell and cytokine responses

**Table 2:** Molecular biomarkers for the detection of CRC.

Clinical use	Subjects	Types	Potential markers
In use	Stool	Protein	Fecal hemoglobin
	Serum	Protein	CEA
		Carbohydrate	CA19.9
Clinical validation	Stool	DNA	K-*ras*
		DNA	*APC*
		DNA	L-DNA
		DNA	*p53*
	Serum	Protein	TIMP-1
Preclinical development	Serum	Protein	Spondin-2, DcR3, Trail-R2, Reg IV, MIC1
		Protein	PSME3
		Protein	NNMT
		Protein	CRMP-2
		Protein	SELDI (apolipoprotein C1, C3a-desArg, α1-antitrypsin, transferring)
		Protein	HNP 1–3
		Protein	MIF
		Protein	M-CSF
		Protein	M2-PK
		Protein	Prolactin
		Protein	CCSA-2, −3, −4
		Protein	MMP-9, −7
		Protein	Laminin
	Plasma	DNA	Septin 9
	WBC	DNA	5-gene panel (CDA, BANK1, BCNP1, MS4A1, MGC20553)

**Table 3:** miRNA related to prognosis of cancer.

Cancers	miRNA	Authors and Ref. nos.
Lung cancer	hsa-let-7	[59]
	hsa-let-7a-2	[60]
	hsa-miR-155	
	hsa-miR-196a2	[61]
	hsa-miR-221	[62]
	hsa-let-7a	
	hsa-miR-137	
	hsa-miR-372	
	hsa-miR-182*	
	hsa-miR-21	[63]
Hepatocellular carcinoma	hsa-miR-125b	[64]
Breast cancer	hsa-miR-21	[65]
Gastric cancer	hsa-miR-21	[66]
Colorectal cancer	hsa-miR-21	[67]
	hsa-miR-106a	[68]
Head and neck cancer	hsa-miR-7d	[69]
	hsa-miR-205	
Pancreatic cancer	hsa-miR-21	[70]
Acute myelogenous leukemia	hsa-miR-181 family	[71]
Chronic lymphocytic leukemia	hsa-miR-1-miR-15a	[72]
Ovarian cancer	hsa-let-7a-3	[73]
Esophageal cancer	hsa-miR-103/107	[74]
